# Emphasizing High Value, Cost-Effective Care in Physical Examination Instruction – A Qualitative Content Analysis of Interviews with Expert Educators

**DOI:** 10.15694/mep.2018.0000064.1

**Published:** 2018-03-21

**Authors:** Paul A. Bergl, Jeanne M. Farnan, Evelyn C.Y. Chan

**Affiliations:** 1 Medical College of Wisconsin; 2 University of Chicago

**Keywords:** Clinical skills, Curriculum development, Hiigh value care, Physical diagnosis, Clinical reasoning

## Abstract

This article was migrated. The article was marked as recommended.

*Introduction*

Physical examination and cost consciousness are critical competencies for medical trainees, but the intersection of these two skill domains is not described. We aimed to elucidate the role of physical examination in providing high value, cost conscious care (HVC) and to explore how clinical skills curricula could integrate principles of HVC.

*Methods*

We conducted a qualitative study of semi-structured interviews with 20 experts in the instruction and clinical applications of physical examination. We identified experts through purposeful sampling and snowball sampling. Audio-recorded interviews were coded using qualitative content analysis. Coded passages were categorized and reported as key themes and recommendations.

**Results**

Experts affirmed physical examination’s indispensable role in clinical reasoning. When integrated with history-taking and additional diagnostic data, physical examination can further the aims of HVC. However, experts noted that the pace and demands of contemporary clinical practice present barriers to the idealized application of physical examination. In turn, participants discussed how to improve clinical skills curricula, both broadly and to promote HVC.

*Discussion*

To advance HVC through physical examination curricula, the clinical relevance of bedside skills needs to be emphasized across the training spectrum. Key strategies include revisiting evidence-based medicine principles and integrating physical examination instruction with teaching clinical reasoning.

## Introduction

As soaring health care expenditures outpace the growth of the American gross domestic product [ Centers for Medicare and Medicaid, 2015], high value care (HVC) has become a curricular priority in medical education [ Ryskina et al, 2014; Korenstein et al, 2016, Patel et al, 2015]. While many factors contribute to overuse of healthcare resources, the culture in medical education likely has played a prominent role [ Emanuel & Fuchs, 2008; Smith & Levinson, 2015]. Accordingly, educational campaigns have been launched to effect cultural change; examples include the American Board of Internal Medicine (ABIM) Foundation’s *Choosing Wisely* initiative [ ABIM Foundation, 2017] and the American College of Physicians and the Alliance for Academic Internal Medicine curriculum in HVC. [ American College of Physicians, 2017] Cost awareness has even become a core competency in systems-based practice in contemporary medical education.[Association of American Medical Colleges (AAMC), 2014].

Performing comprehensive and focused physical examinations are also expected competencies of graduating medical students [ AAMC, 2014]. In the past decade, a task force convened by the AAMC provided a blueprint for today’s clinical skills curricula [AAMC, 2008]. Since this AAMC report, the concept of teaching and assessing learners on a hypothesis-driven physical examination has emerged as an alternative to the traditional approach of teaching head-to-toe examinations [ Yudkowsky et al, 2009]. Other educators have subsequently called for a streamlined ‘core and clusters’ approach in which learners master a foundational basic examination and augment this exam with clusters of maneuvers specific to the clinical context [ Gowda et al, 2014a; Gowda et al, 2014b].

The desire to streamline physical examination instruction stems from a perceived need to eliminate maneuvers of “little clinical or educational value”[ Gowda et al, 2014b] and to impart learners with more effective clinical reasoning skills [ Yudkowsky et al, 2009]. While the hypothesis-driven approach and the core and clusters model both hint at the concept of value, they do not explicitly address how a more selective physical examination allows trainees to deliver HVC. Likewise, HVC curricula to date have not emphasized bedside skills [ Smith & Levinson, 2015; Stammen et al, 2015]. Even the popular Choosing Wisely recommendations for clinical practice rarely mention how physical examination findings can obviate further low-value testing [ABIM Foundation, 2016].

This lack of integration between teaching physical examination and HVC is surprising; physical examination represents one of “the most cost-effective of all tests” [ Schattner, 2012]. When thoughtfully applied in a hypothesis-driven manner, physical examinations should reduce unnecessary follow-up studies, especially when fairly robust evidence for specific examination maneuvers exists [ Simmel & Rennie, eds., 2008; McGee 2012]. Physical examination still helps to establish most medical diagnoses [ Paley et al, 2011, Reilly 2003], and its omission contributes greatly to diagnostic error [ Verghese et al, 2015; Schiff et al, 2009], an enormously costly problem itself [ Newman-Toker et al, 2013]. Conversely, undirected physical examinations may represent low value care themselves [ABIM Foundation, 2016; Mehrotra & Prochazka, 2015] and may uncover false positive findings that in turn can beget cascades of exorbitant testing and even treatment [ Rothberg 2014].

We feel that clinical skills curricula are prime venues to enhance trainees’ provision of HVC [ Bergl et al, 2015], and we sought to understand how clinicians and educators viewed the intersection of HVC and physical examination. Because much of the curricular framework and content for both of these subjects relies on expert consensus [ Corbett et al, 2008a; Cassel & Guest, 2012], and not necessarily on empirical evidence, we examined the opinions of educational leaders in physical examination instruction to explore this novel area of inquiry. The primary aim of this study was to elucidate how experts understood the concept of a *high value, cost effective physical examination.* Additional areas of inquiry included (1.) understanding real-life barriers to using physical examination to help provide HVC and (2.) determining the optimal teaching strategies and content for a curriculum in the high value physical examination. The ultimate goal of this study was to create an idealized curricular model that integrated clinical skills with principles of HVC.

## Methods

### Study Overview

We designed a qualitative study based on interviews with national experts in physical examination. We used semi-structured, partially scripted interviews to gather perspectives of those teaching physical examination and to interpret potential meanings of a *high value physical examination.* Our interview data was analyzed with conventional qualitative content analysis using an inductive approach [ Cho JY, 2014; Hsieh & Shannnon 2005; Teherani et al, 2015; Elo & Kyngas, 2008]. To direct interviews, we created a scripted interview guide (Appendix A) consisting of open-ended questions that corresponded to themes that we had previously identified in the literature [ Bergl et al, 2015]. The institutional review board (IRB) at the Medical College of Wisconsin (MCW) deemed this study exempt from IRB oversight - project #24436.

### Recruitment

Potential subjects were initially identified through purposeful sampling from the professional networks of the study team and from a review of contemporary literature on teaching physical examination. To account for regional variations in health care expenditures and its impact on trainees [ Arora, 2012], we sampled by primarily focusing on geographic diversity of subjects. After generating a list of initial contacts, additional subjects were recruited by snowball sampling. Inclusion criteria required that subjects had at least one of the following qualifications: (1.) authored at least one peer-reviewed publication on physical examination instruction or clinical skills education, (2.) authored or contributed to a physical examination textbook, (3.) actively participated in the AAMC’s Directors of Clinical Skills (DOCS) group, or (4.) directed a physical diagnosis or clinical skills course at their own institution. For subjects identified by snowball sampling, we searched Medline and reviewed online biographies on public websites to determine eligibility for participation. All subjects were invited to participate by email and were offered a $30 Amazon.com gift card for participation.

### Development of Interview Guide

Our interview guide focused on four areas of inquiry: defining physical examination’s role in providing HVC, identifying ideal content for a medical school curriculum on high value physical examination, discussing optimal delivery of such a curriculum, and describing barriers to using physical examination in a cost-effective manner in daily practice. The four key questions to our guide were directly informed by our previously published opinions on the interview topics [ Bergl et al, 2015] and one of our investigator’s (J.F.) involvement in DOCS, a national organization dedicated to enhancing clinical skills curricula.

In developing our guide, we deliberated over whether to provide a definition of *high value* and to explicitly discuss cost-effectiveness. Recognizing that the term *high value* is variably interpreted and does not always imply cost-effective [ Blumenthal-Barby 2013; Porter 2010], we ultimately agreed to begin the interview with an open-ended question including both terms: *value* and *cost* (Appendix A). Prior to contacting subjects, this interview script was piloted with two clinician-educators at MCW. (These preliminary interviews were not included in our final analysis.) Piloting the interview did not result in any major revisions. As we interviewed subjects, the interview guide underwent two minor revisions that provided clearer follow-up probe questions; the four key questions used to guide the interview were not altered at any point.

### Data Collection

Between August 2015 and January 2016, our primary investigator (P.B.) conducted semi-structured telephone interviews with individual participants. While the interviewer had previously made brief contact with several of the subjects prior to the study period, there were no ongoing, substantive professional relationships between the interviewer and interviewees at the time of the study. All subjects provided verbal consent to participate. Interviews were recorded using a digital device and were subsequently transcribed. Transcripts were stored on a secure internal server at MCW and imported into the qualitative research software package NVivo (Version 10.0, QSR International, Melbourne) for analysis.

### Data Analysis

We analyzed our data using conventional qualitative content analysis and inductive open coding [29, 31]. In our approach, we reviewed subjects’ responses to the open-ended interview questions for key statements and experiences, and we used these key passages to formulate an initial set of codes. Our coding was open and grounded in the themes that emerged from the interview transcripts. In other words, even though we anticipated that our four key interview questions might form the four main domains in our coding scheme, we did not define or apply codes a priori.

We developed our coding scheme iteratively while interviews were ongoing. After the first ten interviews, two investigators (P.B., E.C.) met to review a sample of 4 de-identified transcripts. Prior to this meeting, both team members had applied open coding to these 4 transcripts using NVivo. We (P.B. and E.C.) then generated a preliminary coding scheme and initial list of clusters and codes to facilitate future analyses. The remaining interviews were coded independently by the same investigators with regular meetings to discuss discrepancies in coding, to describe emerging themes, and to determine the need for codebook revisions. A third investigator (J.F.) periodically reviewed coded interview transcripts, adjudicated disagreements about the coding scheme, and helped formulate the final codebook.

Prior to closing the study, the study team reviewed coded passages from the first 17 interviews to assess for theme saturation. At a team meeting, we explored whether the codebook had evolved substantially during the study period, a finding that would suggest themes were still emerging. We found no evidence of new themes when comparing the codebook to previous iterations and concluded theme saturation had been achieved.

## Results

Twenty physicians participated in interviews and represented 19 unique medical schools. Subjects had a mean of 24 years of clinical experience since medical school graduation. Additional demographic information is available in Table 1.

**Table 1.** Subject characteristics

Gender			
	Male		12 (60%)
	Female		8 (40%)
***Specialty***			
	Internal medicine	General internist	13 (65%)
		Subspecialist	3 (15%)
	Medicine-Pediatrics		1 (5%)
	Pediatrics		1 (5%)
	Family medicine		1 (5%)
	Emergency medicine		1 (5%)
***Practice setting***			
	Inpatient and outpatient		12 (60%)
	Exclusively outpatient		4 (20%)
	Exclusively inpatient		4 (20%)
***Geographic area of practice***			
	Northeast		7 (35%)
	South		3 (15%)
	Midwest		5 (25%)
	West		5 (25%)
***Evidence of expertise***			
	Previous peer-reviewed publication(s)		12 (60%)
	Educational leadership role (local or national)		19 (95%)

As interviewees discussed the idea of using physical examination to promote HVC, three major themes emerged: (1.) Physical examination has value in contemporary clinical medicine that can in part be captured by economic analysis but that often defies a quantifiable cost benefit. (2.) Various practical factors impede clinician’s abilities to use physical examination to provide HVC, and many of these are attributable to the physicians themselves. (3.) Opportunities to improve physical examination instruction abound -- both generally and within the framework of HVC - and specific areas would merit more attention in a curriculum seeking to integrate these two subjects. These themes are further explored in the following sub-sections and in Tables 2- 5. Direct quotations are in italics.

## Physical examination’s continued relevance in clinical medicine

Though prompted to define a “cost-effective, high value physical examination”, subjects tended to discuss the value of physical examination more broadly. Nonetheless, subjects cited physical examination’s critical role in clinical decision-making and diagnostic reasoning as having significant economic implications. Interviewees suggested multiple other ways in which physical examination could contribute to HVC as elaborated below and as represented in Table 2.

**Table 2.** How physical examination can advance high value care in clinical practice

Value added by physical exam	Exemplary applications
Enhancing diagnostic reasoning	• Using physical exam to test diagnostic hypotheses • Having command of evidence basis and test characteristics (e.g. sensitivity, positive likelihood ratio) of bedside examination maneuvers • Integrating clinical history and physical findings to establish probability of a disease condition
Reducing downstream costs	• Ordering fewer follow-up diagnostic tests after careful examination
Assessing impact of disease	• Determining functional impact of a complaint • Assessing whether patients’ complaints are severe enough to require more than conservative treatment
Establishing clinical diagnoses	• Relying on clinical findings to establish largely clinical diagnoses e.g. rheumatoid arthritis, cellulitis • Recognizing face validity of bedside examination when evidence for utility is underdeveloped e.g. the newborn evaluation
Fostering connections between patient and physician	• Building trust and enhancing communication through physical connection • Providing reassurance to patients • Taking advantage of the healing touch
Adding joy to practice	• Keeping clinical practice stimulating • Finding satisfaction in the “detective work” of investigating patients’ complaints
Providing fringe benefits	• Using technology to enhance senses at the bedside e.g. point-of-care ultrasound, pan-optic ophthalmoscopy • Maximizing success of blind bedside procedures e.g. arthrocentesis • Using validated aspects of the screening exam e.g. blood pressure measurements

*Enhancing diagnostic reasoning.* Participants almost universally noted how physical examination can test hypotheses and refine probabilities of disease. By extension, participants saw indirect economic benefits from skilled history-taking and physical examination.

*When I tried to structure our curriculum, I spent a lot of time with the Rational Clinical Examination series in JAMA... thinking about which parts of the physical exam have test characteristics that would make them diagnostically valuable. And if they’re diagnostically valuable, then theoretically they should have the ability to lower the number of unnecessary tests we’re ordering to ostensibly confirm or rule out a diagnosis.*

Directly reducing costs. Participants also underscored physical examination’s role in limiting testing that is already widely believed to be unnecessary and costly.

*An easy [example] that comes to mind on the outpatient side is evaluation of low back pain.. you know, super common complaint. There’s a lot of good information about what components of the history and exam are high yield. I think there’s a lot of potential savings there... I think maybe we could avoid a lot of unnecessary imaging with all its undesirable downstream effects.*

Assessing the impact of disease on the patient. Participants pointed out that physical examination can be used to assess the functional impact of a patient complaint and to monitor trajectory of illness and response to treatment.

*One [scenario] is just following somebody’s diuresis with heart failure. What things change and what tells you they’re actually getting better? You don’t really have to ‘re-x-ray’ them or ‘re-echo’ them to make sure they’re getting better.*

*Establishing clinical diagnoses.* Though most participants referenced available resources that provide test characteristics for physical findings [ Simmel & Rennie, eds., 2008; McGee 2012], several noted that not all value in physical examination can be quantified. These scenarios included diagnoses which are highly based on clinical features (e.g. cellulitis, rheumatoid arthritis, and decompensated heart failure).

*I think probably the highest value physical examination is one where the findings are actually superior to other diagnostic tests, or in fact, there is no diagnostic test that even exists for that. A classic example would be Parkinson’s disease. That’s probably the best example of cost effective physical examination because it’s [historically been] the only choice.*

Similarly, clinicians had encountered other scenarios in which the evidence is underdeveloped or non-existent, but in which logic suggests unequivocal economic value for physical examination. A pediatrician suggested the newborn examination was the quintessential example in which value had not been explicitly quantified, but she highlighted the critical role of early detection of congenital abnormalities in optimizing newborn health.

Fostering connections. Participants routinely mentioned how they use physical examination to develop connections, to provide reassurance, and to facilitate communication.

*This is a ritual, and it establishes trust.. hands-on [is] a very important connection with the patient.*

*I’m big into some of the nursing literature around perhaps even a therapeutic value of physical exam and laying on [of] hands. Even if the physical exam’s not used diagnostically, there’s some evidence that those who had a physical exam felt better.*

## Factors impairing physical examination’s contribution to clinical practice

Though participants saw physical examination as a core skill in providing high value clinical care, they identified a range of practical limitations and cultural factors that diminish physical examination’s role in providing cost-effective care. As demonstrated in Table 3, barriers were identified in four domains: (1.) the patient-physician interface, (2.) the practice of modern medicine, (3.) the nature of physical examination itself, and (4.) the current state of physical examination instruction. The most frequently discussed factors are further detailed below.

**Table 3.** Factors impairing physical examination’s contribution to high value care

Domain of barrier	Factors encountered in practice	Specific limitations experienced by clinicians and educators
Physician-patient interface	Physician-specific factors	• Inadequate clinical experience • Lack of self-efficacy and confidence in performing physical examination • Improper integration of physical findings within diagnostic reasoning process • Poor recognition of nuances of clinical examination, poor sensory perception
	Patient-specific factors	• Expectations to have bloodwork or advanced diagnostic imaging • Poor mobility, obese body habitus • Anxieties and intolerances to uncertainty
Practice of contemporary medicine	Routines and standards of care	• Algorithmic and protocolized approaches to diagnostic evaluation lessen need for physical examination. • Culture of academic medical centers is to prioritize additional diagnostic testing.
	Availability of technology	• Ease of ordering advanced diagnostic tests results in physicians’ forgoing examination. • Physicians depend on technological surrogate, rather than clinical impression, to establish diagnoses.
	Intolerance to uncertainty	• Physicians’ innate fear of “missing something” prompts further testing. • Laboratory testing and imaging confer additional layers of reassurance to physicians. • Uncommon, high-stakes diagnoses necessitate intolerance to uncertainty e.g. aortic dissection, subarachnoid hemorrhage.
	Practice of defensive medicine	• Diagnostic testing offers more concrete, objective findings than physical examination and thus better protection against litigation. • Diagnostic testing may be viewed as standard of care by litigators and lay people.
	Pace and delivery models in modern medicine	• Fragmented care and desire for clinical efficiency prohibit physicians from observing signs and symptoms over time. • Physicians’ productivity demands and the burden of electronic health record crowd out hands-on elements of care.
Physical examination itself	Incomplete evidence basis	• Evidence for physical examination is not robust enough i.e. studies are too small, have too much interobserver variability, are too old, or are studies of very specific patient populations. • Some aspects of physical examination have not been studied at all.
	Variable value by scenario	• Value of physical examination depends on clinical context and the type of clinician (i.e. generalist vs. specialist) performing it.
Education and instruction in physical examination	Learner factors	• Lack of buy-in, disinterest in learning bedside skills • Distractions including mobile technology and other educational priorities
	Instructor-related factors	• Availability of skilled bedside teachers • Inflexibility and intolerance to curricular innovation
	Curricular factors	• Breadth of curriculum, number of skills needed to master • Lack of formal assessments of physical examination; assessments not optimized to grade technique or integration with clinical reasoning
	Institutional culture and learning environments	• Poor administrative support for curricular innovations; lack of investment in clinical skills curricula • Decreasing emphasis on humanities in premedical and medical education

The patient-physician interface *.* By far, experts cited physicians themselves as the biggest hindrance to using physical examination to provide HVC. Common themes included a lack of confidence around bedside skills, inadequate training, a lack of clinical experience, and a general decline in observational and perceptive skills at the bedside.

The next most commonly referenced barrier were patients. Physicians referenced the difficulties in performing and interpreting physical examination on patients who were obese, frail, or immobilized. Furthermore, many experts perceived that patients often expect advanced diagnostic testing and have their own intolerance to uncertainty that can only be assuaged with further testing.

The practice of modern medicine. Participants pointed out that routines and clinical guidelines often render physical examination unnecessary in diagnostic evaluations. Many participants also stated that the widespread use of imaging and laboratory studies has negatively impacted physical examination’s value. In turn, access to technology has fed into a reliance on technological surrogates to diagnose disease. The ubiquity of technology also contributed to a stronger sense of efficiency; simply stated by one expert, ‘Sometimes it’s just quicker to order the test.’

At least one expert felt that this focus on increased clinical efficiency and physician productivity has crowded out the more humanistic elements of care such as physical examination.

*The bean counters want to bill more and they want to penalize us if we don’t bill more. The doctors are spending more time with a computer, less with the patients. So, tell me, Where you are going to fit the physical exam [in]?*

Participants voiced concerns about litigation as well, and specifically feared that physical examination may not provide adequate protection against a lawsuit. There was often the sense that diagnostic tests are seen as a standard of care in court and provide additional objective evidence.

*You just order the test because then you’ll have something concrete that you can put in the record - [something] defensible so to speak -- versus just your physical exam.*

Beyond litigation, participants often feared missing diagnoses simply out of concern for the patient’s well-being; advanced diagnostic testing provides reassurance for the provider as well. More importantly, for many uncommon yet high stakes diagnoses, physical examination was felt to be too inadequate to forego further testing.

Physical examination itself. While often touted as a strength, the evidence basis for physical examination was also seen as an impediment. For some participants, the evidence is simply not strong enough to alter clinical routines that include a low threshold for testing. Other concerns included the low interobserver reliability of elements of physical examination and the fact that not all examination maneuvers are validated for every patient complaint. Finally, the validity of the evidence - and therefore the impact of the exam - varies across cases, specialties, and practice settings.

*I think evidence-based physical examination is a little tricky because a lot of times, the indications for the maneuver itself might be different than the indication you’re intending to use it for. So, it’s not that a maneuver has specific test characteristics, [but] it’s in what settings, and for what kind of diagnostic indications is that particular maneuver useful.*

Physical examination instruction. Finally, experts discussed a number of fundamental problems in the existing educational infrastructure that need to be addressed before physical examination can be used to advance HVC ( Table 3).

Teaching strategies and content for a high value physical examination curriculum

Specific clinical areas to teach *.* Participants emphasized problem-focused examinations over an organ-based approach, particularly when discussing how to teach HVC. Approaches to teaching problem-based examinations included focused examinations targeted to a chief concern, clinical assessments (such as intravascular volume status, presence or absence of inflammatory arthritis), clinical diagnoses (e.g. heart failure). Experts rarely mentioned specialized or eponymic maneuvers to enhance diagnostic yield. Topics suggested at least twice appear in Table 4. Overall, the musculoskeletal and neurological examinations were the most frequently referenced organ systems. Participants noted that complaints in these organ systems often generate costly imaging studies that infrequently change management.

**Table 4.** Commonly suggested topics for a high-value physical examination curriculum

Chief Concerns	Number of participants suggesting ^*^
Pain in large joint (knee, hip, shoulder, ankle)	14
Back pain	11
Headache	8
Dyspnea / Shortness of breath	7
Syncope, dizziness, lightheadedness	7
Neck pain and cervical radiculopathy	5
Chest pain	5
Pelvic pain	3
Head trauma	3
Cough	3
Altered mental status	2
Abdominal pain	2
**Specific Clinical Findings**	
Heart murmur	8
Jugular veins	6
Lung percussion and tactile fremitus	3
Retinal/funduscopic exam	2
Cirrhosis and complications (e.g. ascites)	2
Peripheral pulses	2
Peripheral edema	2
Heart gallops	2
Abnormal gait	2

^*^ *Tallies reflect how often chief concerns and clinical findings were explicitly suggested as teaching topics - and not simply mentioned in passing during interviews.*

Teaching strategies. Discussants described how common teaching strategies in physical examination could be adapted to also promote HVC. These included traditional didactic activities like lectures and classroom-based group work, using simulation and standardized patients, and practicing clinical skills on real patients with real pathology. Several participants advised incorporating actual costs of diagnostic testing into didactic and simulation activities.

Our experts also identified how clinical skills curricula could better incorporate HVC principles. First, such curricula should emphasize the clinical relevance of physical examination by highlighting its utility, openly discussing its evidence basis, and acknowledging its limitations. Participants also pointed out that physical examination’s role in clinical reasoning needs to be taught across the training continuum i.e. beyond the preclinical medical school curriculum. Ideally, formal assessments would complement efforts to integrate clinical reasoning with high value care, and experts recommended both formative and summative assessments of these clinical skills. Finally, experts suggested developing master teachers adept in bedside diagnostic skills and the tenets of HVC. Details of these proposed improvements are outlined in Table 5.

**Table 5.** Proposed improvements to physical examination curricula to emphasize high value care

Proposed improvements	Specific strategies to advance high value care
Discussing clinical relevance	• Integrating physical examination instruction with teaching clinical reasoning • Upholding principles of evidence-based medicine and Bayesian approaches to diagnostic evaluation (e.g. using likelihood ratios) • Comparing diagnostic performance of physical examination to other commonly performed diagnostic tests • Acknowledging limitations of physical examination in practice • De-emphasizing and eliminating maneuvers with little diagnostic value
Formally assessing bedside skills	• Holding advanced learners accountable for physical examination skills with graded evaluations • Observing trainees using physical examination in actual clinical practice • Providing both formative and summative assessments to trainees • Moving beyond physical examination “checklists” in objective structure clinical examinations (OSCE’s) by assessing technical competence and clinical reasoning
Optimizing timing of content delivery	• Revisiting clinical reasoning concepts longitudinally throughout training • Providing mentored practice in physical examination on clerkships and during residency
Learning from master clinicians	• Demonstrating how to establish patient-physician rapport during physical examination • Advancing physical examination skills by interfacing with subspecialists (e.g. learning detailed knee examination from orthopedist or sports medicine physician) • Dissecting diagnostic thought-processes of master clinicians

## Discussion

Expert clinicians and educators view clinical and diagnostic reasoning as the key links between teaching HVC and physical examination instruction, and many of our findings reflect this overarching theme. While physical examination adds value to practice in many ways, the predominant message was that physical examination is critical to hypothesis testing and clinical assessments. Educational strategies to integrate HVC with physical examination instruction should include reinforcing trainees’ reasoning skills and discussing the evidence basis of physical diagnosis. Our subjects cited numerous factors that impair physical examination’s contribution to contemporary medical care, and any curriculum connecting HVC to physical examination would need to acknowledge these limitations.

Interviewees preferred to focus more generally on the value of physical examination and often avoided discussing costs outright. These findings are unsurprising given the ambiguous nature of the term *high value care* [ Blumenthal-Barby, 2013; Porter, 2010; Riggs & Knight, 2017]. In agreement with other experts’ opinions, our experts saw physical examination enhancing HVC by facilitating patient-physician connections and as a low-cost monitor of treatment responses or disease progression [ Zaman et al, 2016].

We did not further define *value,* and this approach may have neglected the patient’s perspective. Patients may find value when being examined leads to a positive subjective state, such as reduced anxiety and reassurance. [ Blumenthal-Barby, 2013] On the other hand, some elements of physical examination, such as the rectal examination, are uncomfortable. If these elements provide little objective value, (i.e. they have limited diagnostic yield) and create a negative subjective state (i.e. pain and embarrassment), then patients may find more *value* in other diagnostic approaches. Clearly understanding value in health care as a relationship between outcomes per unit of cost [ Porter, 2010] may trivialize other ways that physical examination adds or subtracts value to the patient encounter, and focusing exclusively on using physical examination to contain costs could send the wrong message about HVC [ Riggs et al, 2017].

Viewed through the lens of HVC, historical approaches to teaching physical examination may need modernization. Consistent with the “core and clusters’ and hypothesis-driven approaches that have recently appeared in the literature, [ Yudkowsky et al, 2009; Gowda et al, 2014b] our experts advocated for more pragmatic curricula that prioritized clinical reasoning and hypothesis testing. They also called for longitudinal development of skills rather than relying on the current model of a defined preclinical curricula followed by inconsistent reinforcement throughout clinical rotations. While discussants were open to incorporating HVC into teaching physical examination, they discussed a number of barriers that already constrain clinical skills curricula, such as faculty availability and the breadth and depth of the curriculum. These larger issues might need to be resolved before fully integrating HVC. On the other hand, our work aligns with recent efforts to streamline clinical skills curricula by establishing a core curriculum [ Gowda et al, 2014a] and determining optimal timing for introducing more advanced skills. [ Corbett et al, 2008b; Harin et al, 2013] Finally, our work suggests that trainees should learn clinical skills from instructors versed in both bedside diagnostic skills and HVC; as reflected by our experts’ perspectives, teachers adept in clinical reasoning would fulfill this role.

**Figure 1.**
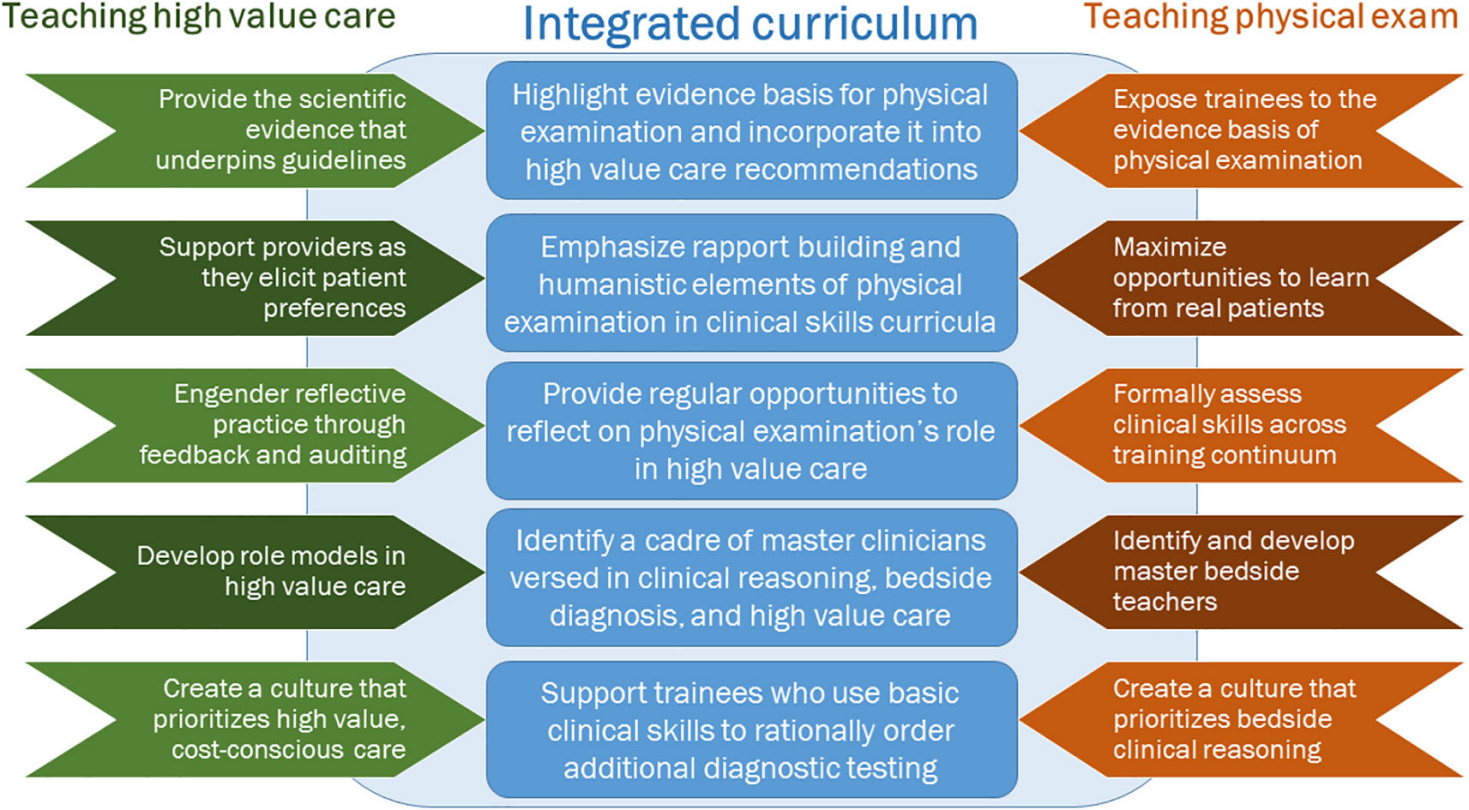
Framework for an integrated curriculum in teaching high value care and physical examination

Our work highlights specific chief concerns and clinical findings ( Table 4) that could be priorities for streamlined curricula, and this study provides a novel framework for teaching physical examination skills. We did not ask subjects to elaborate on why specific chief concerns or findings were mentioned though experts gravitated toward conditions often recognized in other high value educational campaigns. [ ABIM Foundation, 2017] Items in Table 4 may simply reflect familiarity with other HVC curriculum. Accordingly, rather than using Table 4 as the sole blueprint for advancing physical diagnosis curricula, we would suggest a more integrated approach based on our findings as shown in Figure 1.

Our findings also add to the literature on how to better teach HVC. Educational strategies suggested by our experts overlap significantly with recent calls to incorporate more experiential learning and foster a culture that supports trainees in providing HVC [ Smith & Levinson, 2015]. Thus, integrating HVC with physical examination instruction may have a synergistic effect in advancing learning in either domain. Consistent with other studies [ Colla et al, 2016; Zikmund-Fisher et al, 2017; Bishop et al, 2017; Kanzaria, 2015], we found that physicians’ perceptions of patient expectations and fear of liability diminish physical examination’s role in providing cost-conscious care. However, our experts more frequently implicated the physicians themselves and the clinical environments in which they work as drivers of overuse of low value services. Leaders in HVC should address the other barriers noted by our interviewees ( Table 3), and educators should strive to impart trainees and clinicians with greater confidence in their bedside skills. We found that many physicians still view trust-building as one of the primary roles of performing a physical examination, a view that may be shared by patients as well [ Kadakia et al, 2014]. Educators developing HVC curricula may wish to highlight this role in particular. Trainees might even be given specific strategies to capitalize on this valuable aspect of the bedside examination.

There are several key limitations to our study. First, many subjects were identified by their expertise in curriculum development in clinical skills, not by their command of HVC. Because of this academic focus, and perhaps due to the ambiguity of the term *high value*, subjects gravitated toward discussing how to best teach physical examination. When prompted to recommend content for a high value physical curriculum, subjects often had difficulty generating specific topics extemporaneously. Some clinical areas also engendered controversy; for example, echocardiographic evaluation of systolic heart murmurs was referenced as both a quintessential example of overuse and also as an example of necessary care because of the limitations of the bedside assessment. Additionally, as participants mostly were internists by training, clinical and educational expertise from other specialties were highly under-represented. Thus, suggestions on curricular content ( Table 4) should not be interpreted as a definitive guide to a physical diagnosis curriculum for advancing HVC. Furthermore, all subjects held medical school faculty appointments at the time of their interviews, so their opinions may not reflect practice in non-academic settings. Because most of our subjects work directly with trainees, their perceptions of what “other physicians” do are likely skewed. On average, subjects had been in practice for 24 years, and generational differences may also be relevant in this type of study. Further studies including other medical specialties, physicians in non-academic settings, younger physicians, and non-experts would strengthen our conclusions. Given the conversational nature of semi-structured interviews, we also cannot exclude bias introduced by the interviewer during questioning. Because we began coding prior to conducting all the interviews, it is possible that subsequent interviews and analysis were colored by the investigators’ impressions of preliminary data. In other words, we may have introduced ascertainment bias, or finding what is expected to be found. Finally, our sample size may not have allowed for complete thematic saturation. Even during our last few interviews, relatively novel perspectives were shared. However, the research team agreed these emerging ideas still fit within existing codes, and thus we believe our results sufficiently capture major themes that answer our primary research questions.

## Conclusion

In summary, our research offers key implications for clinical skills curricula and the HVC movement. It argues for physical examination instruction and assessments to more frequently incorporate elements of clinical reasoning. Even advanced learners may need additional instruction in physical examination as these learners possess the scaffolding onto which advanced diagnostic reasoning and HVC can be added. To substantiate physical examination’s role in HVC in clinical practice, learners will need to receive mentorship from master clinicians fluent in both bedside skills and diagnostic reasoning. Educators should underscore all valuable elements of physical examination and should point out that not all of this value is quantifiable. These teachers will also need to acknowledge the myriad limitations of physical examination encountered in daily practice. Finally, clinical skills curricula can borrow approaches from the HVC movement to impart learners with a commitment to HVC as outlined in Figure 1.

## Take Home Messages

Physical examination and high value care are two major areas of competency in medical trainees, but these competencies are often taught in siloed curricula. We believe that integrating high value care into teaching physical examination could make clinical skills curricula more practical. However, the available literature provides little guidance on how to achieve that aim. This paper summarizes how expert educators conceive of a “high value physical examination,” and it provides a roadmap for marrying clinical skills curricula with efforts to teach high value care. Ultimately, expert clinicians and educators view clinical and diagnostic reasoning as the key links between teaching HVC and physical examination instruction.

## Notes On Contributors

• Dr. Bergl is a critical care medicine fellow in the Division of Pulmonary, Critical Care, and Sleep Medicine, Medical College of Wisconsin, Milwaukee, WI. At the time of this study, he was assistant professor in the Division of General Internal Medicine, Medical College of Wisconsin.
• Dr. Farnan is an associate professor in the Section of Hospital Medicine, University of Chicago, Chicago, IL where she is Director of Clinical Skills Education at the Pritzker School of Medicine. She is also Assistant Dean for Curricular Development and Evaluation.
• Dr. Chan is an associate professor in the Division of General Internal Medicine, Medical College of Wisconsin, Milwaukee, WI.

## Appendices

### Appendix A- Interview Guide

#### Opening

Dr. ____, I want to thank you for your time and participation in this interview. I hope our conversation will help improve the way we teach physical examination and advance physical examination’s role in contemporary medicine.

#### Statement of Purpose

As I mentioned in our email correspondence, I am leading the design of a physical examination curriculum that explicitly emphasizes the high value and cost-effectiveness of bedside diagnostic skills.

We will be using this curriculum to teach a course for fourth-year medical students at the Medical College of Wisconsin entitled “Advanced Physical Diagnosis” and will also be integrating aspects of the curriculum into our Ambulatory Medicine Clerkship.

However, I am hoping that conversations with experts like you will lead to a robust, expert-derived curriculum adaptable to wider audiences. For example, such a curriculum could be extremely useful to residency programs, other health professional training programs, and even faculty development efforts.

#### Consent

Before we begin, I need to obtain some permissions from you. (Start audio recorder).

• Do you agree to have your *de-identified* responses published or otherwise disseminated? De-identification means that your name will not be attached to any of your responses.
• Do you consent to have the rest of this conversation audio-recorded?

OK, thank you. If at any point you’d like me to stop recording or decide you no longer want to participate, let me know.

#### Background

There is a lot of talk these days about reducing our costs of providing care, and over-testing seems to come up frequently. Now I know that over-testing has many causes, but it seems to me that really focusing on improving basic physician skills like the physical exam could help reduce over-testing - which is where I want to focus my curriculum.

We will have four main areas to discuss: What a high value physical exam means, what you would teach third and fourth year students about the “high value” exam, how you’d deliver this curriculum, and what barriers you see to using a high value exam in real-life practice.

Feel free to ask clarifying questions if I am unclear.

#### DEMOGRAPHICS:

Years in practice _____________________________ Specialty: _____________________

Setting: ______________________________ Educator/pub experience: ______________

Age: _____ Race: ______ Gender: _______

***Main Question 1:*** When I say a “high-value, cost effective physical exam,” what comes to mind?

#### Follow-up questions:

• Besides ____________, can you see any other ways in which physical examination is high value?
• What is the physical examination’s role in providing high-value, cost effective care?
• How would you define a “highly valuable” physical exam maneuver?
• Are there settings in which its value changes?

***Main Question 2:*** What would you include in a high-value, cost-effective physical examination curriculum for fourth-year medical students?

#### Possible Follow-Up Questions

• Think about certain scenarios or patient complaints that seem to prompt diagnostic testing that could be avoided with better physical exams. Now what comes to mind? (Give my own examples if needed.)

• Are there certain patient complaints? How about from an organ systems approach? And how about from the perspective of, “I can’t believe my colleague orders/ordered an advanced diagnostic test in this situation”?
• Let’s put it this way.. If a patient called you after hours with a complaint, in what scenarios would you feel like, “OK, I just need to see you to figure this out”?
• How would the curricular content for students be different than what you would teach interns or residents?
• Would you focus on
  • Knowledge acquisition?
  • Skill acquisition?
  • Improving confidence in diagnostic reasoning?
  • Or something different altogether?

***Main Question 3:*** How would you deliver the content of a “high value physical exam” curriculum?

#### Possible Follow-Up Questions

• What methods of delivery would you use to teach students a “high value physical exam” curriculum?
• In what setting would you teach this? (If prompts needed: Bedside? Classroom? Virtual visits?)
• Do you need actual patients?
• What might you do differently to emphasize the high value and cost-effectiveness of physical exam?

***Main Question 4:*** What do you think are the barriers to using physical exam as part of cost-effective, high-value care in real clinical practice?

#### Possible Follow-Up Questions

• What are the barriers in your clinical practice? How about in your educational leadership roles?
• *Domains of barriers to prompt:*
  • Learner barriers?
  • Clinical?
  • Administrative?
  • Cultural?
  • Institutional?
• Are you in using your own physical examination to reduce wasteful practices in medicine? Are there areas where you yourself could improve?

#### General Probes

**Table:**

Continuity probes	Clarification probes	Example probes
*Tell me more about ____* *You were discussing... Can you expand on that idea?*	*How would ______?* *Why do you say ____?*	*Can you give me an example of that?* *How would that look in practice?*

***Closing:*** Is there anything else we haven’t discussed or I haven’t considered that you wanted to mention?
