# Supplementary material for: Emphasizing High Value, Cost-Effective Care in Physical Examination Instruction – A Qualitative Content Analysis of Interviews with Expert Educators
Source: MedEdPublish (2016). 2018 Mar 21;7:64. [Version 1] doi: 10.15694/mep.2018.0000064.1 (PMC10711970; doi:10.15694/mep.2018.0000064.1)
Supplement: Peer review history

# Reviewer report: Reviewer response for version 1

**DOI:** 10.21956/mep.19534.r28922

**Published:** 2019-05-09

**Authors:** Ken Masters

**Affiliations:** 1 Sultan Qaboos University

This review has been migrated. The reviewer awarded 5 stars out of 5 The paper is a very well-written and interesting qualitative study looking at emphasizing High Value, Cost-Effective Care in physical examination instruction. The paper begins with a strong review of the relevant literature, and then goes on to describe the very well-conducted qualitative study. The paper shows the detailed complexity of the issues, and the many different facets that will need to be addressed in solving the problems. I was particularly impressed with the amount of detail in the results, but presented in such a way that the overall picture is never lost, and the coherent analysis and discussion regarding these results, including the extensive review of these results in light of the literature and the development of the very useful framework in Figure 1.I highly recommend this paper.

Reviewer Expertise:

NA

No decision status is available

# Reviewer report: Reviewer response for version 1

**DOI:** 10.21956/mep.19534.r28923

**Published:** 2018-03-22

**Authors:** Tripti Srivastava

**Affiliations:** 1 Datta Meghe Institute of Medical Sciences Deemed University

This review has been migrated. The reviewer awarded 5 stars out of 5 A very well articulated, well executed and well presented manuscript! The authors have brought forth the need vis-a-vis the present scenario regarding a vital competency for any health professional - the clinical reasoning skills for high value care by physical examination. The qualitative approach is robust and the data analysis is worth to learn not only about the findings but the manner in which it should be analysed and presented. 'Framework for an integrated curriculum in teaching high value care and physical examination' within the manuscript is a splendid suggestion that can be adopted in academic hospitals, especially in areas with economic limitations. Highly recommended!

Reviewer Expertise:

NA

No decision status is available
